# Iron: An Essential Element of Cancer Metabolism

**DOI:** 10.3390/cells9122591

**Published:** 2020-12-03

**Authors:** Myriam Y. Hsu, Erica Mina, Antonella Roetto, Paolo E. Porporato

**Affiliations:** 1 Molecular Biotechnology Center, Department of Molecular Biotechnology and Health Sciences, University of Torino, 10126 Turin, Italy; myriam.hsu@unito.it (M.Y.H.); erica.mina@unito.it (E.M.); 2 Department of Clinical and Biological Science, University of Turin, AOU San Luigi Gonzaga, 10043 Orbassano, Italy

**Keywords:** iron, cancer metabolism, mitochondria, iron-sulfur cluster

## Abstract

Cancer cells undergo considerable metabolic changes to foster uncontrolled proliferation in a hostile environment characterized by nutrient deprivation, poor vascularization and immune infiltration. While metabolic reprogramming has been recognized as a hallmark of cancer, the role of micronutrients in shaping these adaptations remains scarcely investigated. In particular, the broad electron-transferring abilities of iron make it a versatile cofactor that is involved in a myriad of biochemical reactions vital to cellular homeostasis, including cell respiration and DNA replication. In cancer patients, systemic iron metabolism is commonly altered. Moreover, cancer cells deploy diverse mechanisms to increase iron bioavailability to fuel tumor growth. Although iron itself can readily participate in redox reactions enabling vital processes, its reactivity also gives rise to reactive oxygen species (ROS). Hence, cancer cells further rely on antioxidant mechanisms to withstand such stress. The present review provides an overview of the common alterations of iron metabolism occurring in cancer and the mechanisms through which iron promotes tumor growth.

## 1. Introduction

Iron is an essential constituent of hemoproteins and iron-sulfur proteins that are responsible for a plethora of systemic functions, ranging from oxygen transport to cell metabolism and DNA synthesis [1]. Research in the field of iron metabolism has been focused on erythrocytes, while accumulating studies highlight the importance of an adequate iron supply in non-erythroid cells. Besides blood disorders, disrupted iron homeostasis has been linked to pathogenesis of metabolic diseases, including diabetes and cancer [2,3]. While a trace amount of iron is essential for cell survival, excessive iron promotes reactive oxygen species (ROS) generation [4,5]. In particular, cancer cells have to adapt to this double-edged sword as they require unphysiological amounts of iron to sustain cell proliferation which, meanwhile, contributes to increasing oxidative stress. The latter promotes deleterious protein, lipid, and DNA modifications and further metabolic changes, thereby selecting cells with most aggressive phenotype for tumor growth. 

### 1.1. Epidemiology Linking Iron to Cancer

Results from large epidemiological studies have concluded that red meat intake is positively associated with liver, lung, pancreatic, breast, esophageal, and colorectal cancers [6,7,8,9,10,11]. The latter has been given particular attention in epidemiology, and high heme content in red meat was found to contribute to colorectal cancer [12,13]. Notably, in vitro data and human meta analyses collectively indicate that meat-derived heme catalyzes the formation of lipid peroxides [14] and nitroso-compounds (notably nitrosyl-heme and nitrosothiols, [15]), which can give rise to sequential formation of reactive species, eventually leading to stable carcinogenic DNA adducts [16]. Besides dietary iron intake, accumulating clinical studies have found a positive correlation of increased systemic iron levels with different types of cancer in humans (reviewed by Torti et al. [3]). Notably, high transferrin saturation (above 40%) was associated with increased risk of cancer in both genders [17]. Patients suffering from iron overload due to hemochromatosis or homozygous β-thalassemia were also reported to present a higher risk of cancer development, especially liver cancers [18,19,20]. Similarly, patients receiving blood transfusions (which increase iron levels) are also more susceptible to develop cancer [21]. In contrast, iron reduction by regular blood donations, or by phlebotomy in peripheral arterial disease patients, has been associated with a lower risk for cancer [22,23].

### 1.2. Systemic Iron Metabolism

Total body iron depends on age, gender, diet, and state of health and typically varies between 3 and 4 g in healthy adults, of which approximately two-thirds is bound in hemoglobin of red blood cells that is continuously recycled by the reticulo-endothelial system (particularly in the spleen and the bone marrow) [24,25]. Dietary iron absorption is relatively poor, as only 10% of ingested iron (from a balanced meal that contains both heme and non-heme iron) is absorbed in the duodenum and generally yields a negligible 1–2 mg of bioavailable iron [24]. After absorption, iron is exported from enterocytes to the blood system through the basolateral membrane transporter ferroportin (FPN), prior to its binding to transferrin, which allows its circulation in the bloodstream [24,26,27]. Of note, transferrin is the predominant iron-binding protein in circulation; other homologues include melanotransferrin, which is lowly expressed on cell membranes [28,29], and lactoferrin, a glycoprotein secreted by exocrine glands and immune cells with iron-sequestrating properties [30]. Nevertheless, both melanotransferrin and lactoferrin are unable to bind to transferrin receptors (TfRs) and have a minor role in iron metabolism [31,32,33]. Indeed, all cells require iron, but the vast majority of iron is used by the bone marrow for erythropoiesis [34]. Unused iron from peripheral tissues is then stored by the liver, which plays a major role in the maintenance of systemic iron homeostasis by secreting hepcidin. Hepcidin is a peptidic hormone which binds to FPN and induces its internalization and lysosomal degradation, leading to reduced circulating iron levels [35,36,37,38]. 

### 1.3. Cellular Iron Metabolism

Since iron is both essential and toxic, iron uptake, trafficking, and storage are tightly controlled cellular processes that maintain homeostasis. In non-erythroid peripheral tissues, transferrin-bound iron (TBI) binds to its receptor (TfR1) and enters into the cells through endocytosis, is reduced to Fe (II) by six-transmembrane epithelial antigen of prostate (STEAP) reductases, and is released from the endosome by dimetal transporter 1 (DMT1) [39,40]. Unlike TfR1, TfR2 shows low affinity to Tf and is mainly expressed in the liver, where it acts as an iron-sensor, regulating hepcidin production [41]. For heme, several proteins (mostly non-selective) are involved in the uptake, including heme carrier protein 1 (HCP1) and clusters of differentiation 91 and 163 (CD91 and CD163), whose expression and role depend on the cell type. Circulating heme can also be imported via endocytosis, requiring heme-responsive gene 1 (HRG1) to release heme in the intracellular space, which can then be degraded by heme oxygenase 1 (HO1) to yield iron [42]. Iron can remain free in the cytosol, constituting the cytosolic labile iron pool (LIP) readily available for catalytic uses, but a major amount enters mitochondria through mitoferrins (MFRN) 1 and 2 in erythroid and non-erythroid cells, respectively [43,44]. Most of the imported iron is used for heme and iron sulfur-clusters synthesis that mainly takes place in the mitochondria. The biosynthesis pathways of both heme and iron-sulfur clusters have been extensively described previously [45,46,47]. The excess of iron in the cytosol is sequestrated by its storage protein ferritin (FT), composed of heavy and light subunits (FTH and FTL) [48]. During storage, iron is oxidized to the ferric state by FTH, while FTL stabilizes the complex, favoring mineralization [49]. Ferritin degradation by a process similar to autophagy (a process termed ferritinophagy), requiring a cargo receptor nuclear receptor coactivator 4 (NCOA4), releases bioavailable iron back to the cytosol [50]. How iron is reduced back to the ferrous state remains to be elucidated.

Intracellular iron homeostasis is mainly maintained by iron-regulatory proteins (IRP) 1 and 2, which regulate iron uptake, storage, and excretion by post-transcriptional modifications of major proteins involved in iron trafficking [51]. In iron deficiency, IRPs bind to iron-responsive elements (IREs) located in the 5′ or 3′ untranslated region (UTR) of target transcripts. Binding of IRPs to 5′UTR IRE results in translation repression of the mRNA (such as for ferritin and ferroportin), while binding to 3′UTR IRE stabilizes the mRNA and increases translation (such as for transferrin receptor 1) [51]. Although having the same role in the regulation of iron metabolism, the two IRPs function with distinct iron-sensing mechanisms. In iron-replete conditions, IRP1 is an iron-sulfur protein known as aconitase, which exerts enzymatic activity, converting citrate to isocitrate [52], whereas IRP2 is degraded by the ubiquitin-proteasome system after recognition by F-box and leucine-rich repeat protein 15 (FBXL5) [53]. In iron deficiency, the lack of iron-sulfur clusters triggers the functional switch of IRP1 and stabilizes IRP2. 

## 2. Iron as a Catalyst of Cancer Metabolism

### 2.1. Hemoproteins and Cancer 

Heme is a prosthetic group that consists of iron complexed with a protoporphyrin IX ring found in various proteins involved in oxygen binding, metabolism, detoxification, and even signaling [37]. Heme plays a major role in both anti-oxidant reactions. Most catalases (anti-oxidant enzymes that break down hydrogen peroxide to water and oxygen) are heme-containing enzymes that have a ferric protoporphyrin IX (or heme b) in their active sites [54]. It is noteworthy that hydrogen peroxide has toxic effects, as it participates directly in the Fenton reaction, producing hydroxyl radicals, but can also play the role of a messenger, activating inflammatory and apoptotic pathways [55]. Increased catalase expression has been reported in several cancer cell types [56], and recent meta-analyses revealed a correlation between a specific polymorphism of catalase and prostate cancer [57,58]. Besides antioxidant defense, heme also can participate in oxidative stress and inflammation. For instance, nitric oxide synthase (NOS) requires heme as a cofactor to produce NO from L-arginine. Inducible NOS (iNOS or -NOS2) isoform has been commonly found upregulated in tumors, where NO is viewed as an oncogenic molecule that promotes a wide range of pro-tumoral effects, including genome instability, cell invasion, and angiogenesis [59,60]. Importantly, several studies have reported that NO can inhibit M1 macrophage polarization and T-cell differentiation, resulting in immunosuppression [61]. On the contrary, it is widely known that NO generated by immune cells can exert direct anti-tumor effects in animal models [62]. The multifaceted controversial roles of NO in cancer, that are likely dependent on its concentration, have been increasingly acknowledged in recent years.

Similar to the effects exerted by NO, prostanoids (prostaglandins and thromboxane) are arachidonic acid-derived lipophilic signaling molecules that mediate inflammation and vasodilation. Their syntheses are catalyzed by cyclooxygenases COX1 and COX2, which require a heme prosthetic group as well [63]. Both isoforms, especially the second, have been extensively investigated for their role in cancer-related inflammation. COX2 is an inducible form that has been associated with excessive inflammation and carcinogenesis along with increased angiogenesis, tissue invasion, resistance to apoptosis, and immune defense [64,65]. Of note, patients under COX2 inhibitor use presented reduced risk for colon, breast, and lung cancers [65,66,67,68]. However, further investigation is necessary to understand whether iron contributes directly to the pro-tumoral effects of COX and NOS.

### 2.2. Iron-Sulfur Proteins in Cancer

Iron-sulfur clusters (ISCs) are inorganic complexes formed by two or more atoms of iron and sulfur from cysteine residues in proteins. As both iron and sulfur can donate or accept electrons, the resulting coordination presents a wide range of oxidation states [69,70]. Hence, ISCs represent versatile cofactors on which many reactions rely. Several components of its biosynthetic pathway, including cysteine disulfurase (NFS1), frataxin (FTX), and glutathione, have been shown to foster tumor progression and resistance to therapies in different models [71,72,73]. However, frataxin was also reported to induce mitochondrial oxidative metabolism in colon cancer cells, thereby suppressing tumor growth [74]. 

A recently characterized class of iron-sulfur proteins, named CDGSH iron-sulfur domain-containing protein (NEET), has also gained interest in the field of cancer. In non-malignant cells, NEET proteins were shown to be involved in remarkably broad processes, including autophagy, fatty acid metabolism, and insulin secretion [75,76,77]. MitoNEET and nutrient-deprivation autophagy factor-1(NAF1) have been reported to predict negative prognosis in breast, liver, pancreatic, and cervical cancers [78,79,80]. Coherently, mitoNEET and NAF1 promoted tumor growth by inducing mitochondrial oxidative metabolism and resistance to autophagy in breast cancer cells [81,82,83].

Interestingly, iron availability directly regulates mitochondrial biogenesis and oxidative metabolism [84]. Indeed, both heme and ISCs are essential to mitochondrial function as they enable several steps of the tricarboxylic acid (TCA) cycle and electron transport chain (ETC). 

In humans, two reactions of the TCA cycle require ISCs: succinate dehydrogenase (SDH, also complex II of the ETC) and aconitase (ACO) [85]. Deficient expression or activity of these enzymes typically denotes mitochondrial dysfunction that has long been linked to tumorigenesis. Aconitase catalyzes the second step of the TCA cycle, which consists in the isomerization of citrate to iso-citrate via cis-aconitate formation. The conformation of aconitase changes according to the ISC and determines its enzymatic activity: liaison with a [3Fe-4S] cluster is found in the inactive form, while the acquirement of an additional iron atom forming [4Fe-4S] activates the enzyme [86,87]. Decreased mitochondrial aconitase ACO2 has been reported in gastric cancer, wherein it predicted poor prognosis, and in human breast tumor biopsies as well as breast cancer cell lines [88]. In addition, the induction of ACO2 enhanced mitochondrial oxidative metabolism and sustained ROS production, leading to reduced breast cancer cell proliferation [88]. A cytosolic isoform, more commonly termed iron-regulatory protein 1 (IRP1) or iron-responsive element binding protein (IRE-BP), exerts the same catalytic function but serves as a major iron-sensor and mRNA-binding protein, as discussed above [89]. 

Succinate dehydrogenase (SDH) catalyzes the oxidation of succinate to fumarate, which is then hydrated by fumarate hydratase into malate. Mutations inducing SDH-subunit B (SDHB) loss of function or deficiency have been shown to cause the development of paragangliomas, pheochromocytomas, and gastrointestinal and renal cancers [90,91,92,93,94]. Furthermore, SDHB-silencing was sufficient to induce ROS generation and metabolic switch, which increased cancer cell proliferation and tumor growth in vivo [95,96]. Indeed, excessive levels of succinate resulting from SDH stabilize hypoxia-inducible factor 1α (HIF1α) and initiate tumorigenesis; hence, it is considered as an oncometabolite [97].

In addition to the enzymes catalyzing the TCA cycle, iron is a step-limiting factor of the ETC, as all four complexes require at least one ISC, heme, or a combination of both to support the electron-transferring activity [98,99]. Consequently, a functional ETC is essential for efficient ATP generation in order to sustain cell proliferation. An impaired ETC is widely known to cause mitochondrial ROS that activate signaling pathways, fostering tumorigenesis [100,101]. Nevertheless, excessive ROS also trigger apoptosis; hence, inhibitors of ETCs have emerged as anti-cancer strategy [102].

Finally, many enzymes involved in DNA replication and repair carry at least one ISC that is essential for the enzymatic activity. The presence of ISCs is required for functional activity of DNA polymerases, glycosylases, and helicases, as well as exonucleases [103,104,105,106]. Importantly, recent findings show that eukaryotic DNA polymerases require an ISC that is essential for stable interactions between different subunits of the multimeric structure and its binding to double-stranded DNA, and lack of this ISC leads to poor fidelity of replication and genome defects [103,107,108]. Furthermore, iron chelation has been shown to inhibit ribonucleotide reductase (RNR), the enzyme that catalyzes the reduction of ribonucleotide to deoxynucleotides, as well as a class of histone lysine demethylases that enable gene transcription of several oncogenes [109,110,111,112]. Similarly, iron is also essential for nucleotide catabolism. Xanthine oxidoreductases (XORs) catalyze the oxidation of hypoxanthine to xanthine and, ultimately, to uric acid, along with the production of superoxide and hydrogen peroxide. The presence of ISCs is required for the catalytic reaction which contributes directly to oxidative stress by producing ROS and reactive nitrogen species (RNS), while uric acid can further trigger inflammation [113]. In fact, increased serum levels of XOR and uricemia are often associated to metabolic disorders, including cancer [114,115]. Inhibitors of XOR, such as febuxostat and allupurinol, have been reported to inhibit cell migration and exert cytotoxic effects on human breast cancer cells and hormone-refractory prostate cancer cells in vitro, respectively [116,117]. Hence, iron availability controls cell proliferation by regulating DNA synthesis, repair, transcription, and catabolism. 

### 2.3. Free Iron as an Enzymatic Cofactor 

Although it is evident that intracellular iron is mostly complexed within heme or ISCs, free iron can also directly act as a cofactor catalyzing enzymatic reactions. A notable example consists of the family of prolyl/asparagyl hydroxylases (PHDs) that catalyze post-translational hydroxylation of hypoxia-inducible factor 1α (HIF1α), resulting in its ubiquitination and subsequent proteasomal degradation. Besides oxygen, this modification is, in fact, dependent on the presence of 2 oxo-glutarate as the substrate and ferrous iron (Fe^2+^) as the cofactor. The requirement of iron in the degradation of HIF1α was first evidenced by the normoxic stabilization of HIF1α upon desferoxamine treatment in vitro, and chaperone proteins PCBP1 and PCBP2 were later identified to mediate the delivery of free iron to PHDs [118,119]. Once stabilized, HIF1α translocates into the nucleus, where it interacts with HIF1β (also known as ARNT) to bind hypoxia-response element (HRE) [120]. In cancer, HIF1α stabilization mediates many malignant properties, including defective angiogenesis and aerobic glycolysis, a phenotype termed the Warburg effect [121]. 

Similarly, deoxyhypusine hydroxylase (DOHH), the enzyme responsible for the last step of hypusine synthesis, requires iron for catalysis [122,123]. Hypusine is a highly conserved amino acid found solely in eukaryotic translational initiation factor 5A (eIF5A). Functional DOHH is, therefore, essential to cellular viability, as mature eIF5A regulates eukaryotic cell proliferation [124]. Interestingly, decreased levels of miR-331-3p and miR-642-5p were found in human prostate tumors overexpressing DDOH, and overexpression of these miRNAs reduced DOHH levels and hindered proliferation in vitro [125]. Likewise, pharmacological inhibition or RNA interference-mediated silencing of DOHH reduced cervical cancer cell proliferation [126].

## 3. Alterations of Iron Metabolism in Cancer

### 3.1. Systemic Iron Alterations in Cancer

Similar to what occurs in microbial infection, iron metabolism is altered at the systemic level in order to withhold iron stores within the host cells to limit uptake by pathogens. Anemia is commonly found in cancer patients and mostly arises from chronic inflammation characterized by iron restriction and decreased erythropoiesis, known as anemia of chronic disease or of inflammation [127]. For instance, the inflammatory cytokine interleukin 6 (IL-6) mediates hepcidin upregulation through the Janus Kinase/Signal Transducer and Activator of Transcription (JAK/STAT) pathway [128,129,130,131], while tumor necrosis factor-α (TNF-α) inhibits erythropoiesis [132]. In vitro studies have further shown that other cytokines, such as interleukin 1-β (IL-1β), also promote hepcidin synthesis [133,134]. Importantly, immune cells, including macrophages and neutrophils, also secrete hepcidin through TLR-4 induction as an innate immune response [135,136,137]. It is noteworthy that bone morphogenic proteins (BMPs), a class of transforming growth factor β ligands whose upregulation has been reported in various cancers, including breast, prostate, and bladder cancers, also induce hepcidin secretion by cancer cells [138]. Consistently, circulating plasma hepcidin is typically high and correlates with disease stage in breast, non-small lung, urothelial, and renal cancers [139,140,141,142,143]. Elevated serum ferritin has also been reported in many types of cancer, including neuroblastomas, lymphomas, colorectal, cervical, and breast cancers, and has been used as both a diagnostic tool and a prognostic factor [144,145,146,147,148,149]. Interestingly, excessive circulating ferritin, which modulates cancer cell metabolism and stimulates cancer cell proliferation, originates largely from tumor-associated macrophages (TAMs) [150]. 

### 3.2. Cellular Iron Dysregulation in Cancer 

**Figure 1:**
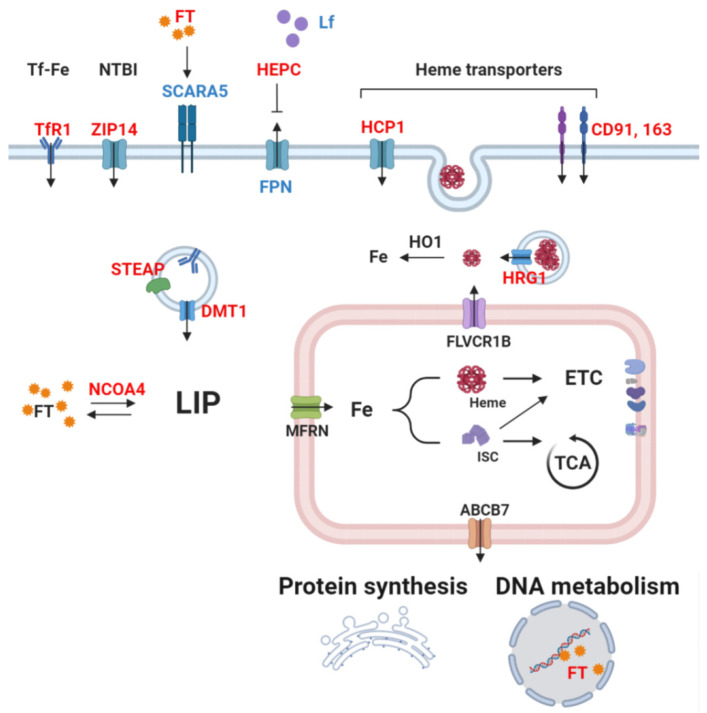
Simplified scheme of cellular iron metabolism in cancer cells. Red: upregulation reported in cancer. Blue: downregulation reported in cancer. Cancer cells commonly show increased uptake of iron by transferrin receptor 1 (TfR1). After binding of transferrin-bound iron (Tf-Fe or TBI) on its receptor, the complex is carried to the intracellular space by endocytosis. Coordination of six-transmembrane epithelial antigen of prostate (STEAP) and divalent metal transporter (DMT1) releases free iron in the cytosol. Circulating free, non-transferrin-bound iron (NTBI) can also be imported by ZIP14. Hepcidin (HEPC) secretion by the liver downregulates ferroportin (FPN) levels, consequently causing iron accumulation in the intracellular space. Cancer cells also deploy other iron-acquisition methods, e.g., via heme importers, including HCP1, CD91, and CD193. Heme-responsive gene 1 (HRG1) allows the cytosolic release of heme imported by endocytosis. Moreover, increased ferritinophagy, and hence the degradation of ferritin–iron (FT) complexes by NCOA4, further increases bioavailable iron. Altogether, these mechanisms lead to elevated labile iron pool (LIP). After incorporation in enzymes’ prosthetic moieties or alone as a cofactor by the mitochondria, iron can fuel the TCA cycle and the electron transport chain or be exported to the cytosol and participate in translational process as well as DNA replication or repair.

Cancer cells display peculiar alterations in iron metabolism that depict an overall increased iron turnover, with an enhanced affinity for iron that could be considered as a hallmark of cancer [151] (Figure 1). TfR1 overexpression has been commonly observed in both cancer cells in vitro and tumor tissues, including glioma, leukemia, and breast, ovarian, prostate, colorectal, and liver cancers [152]. Consequently, high TfR1 levels are typically associated with poor prognosis. In contrast, TfR1 silencing impeded oxidative phosphorylation and proliferation of pancreatic adenocarcinoma cells [153] and decreased breast tumor growth and lung metastases in mice [154]. Interestingly, p53 induction markedly downregulated TfR1 levels and led to cell cycle arrest in human lung cancer cells [155]. Similarly to TfR1, melanotransferrin is notably upregulated in melanoma tissues and, to a lesser degree, in liposarcoma and breast and lung cancers [156]. Moreover, melanotransferrin overexpression is associated with high tumor grade and metastases in human colorectal cancer [157]. Interestingly, despite a minor role in iron metabolism, gene silencing of melanotransferrin markedly reduced melanoma cell proliferation and tumor growth in mice [158]. A soluble form of melanotransferrin characterized to have low efficiency in delivering iron was found to foster melanoma cell migration, invasion, and endothelial cell angiogenesis [29,159,160]. In contrast to its homologues, lactoferrin (Lf) has been shown to possess anti-tumoral activity, with abnormally low levels found in several cancers. In particular, hypermethylation of Lf promoter was shown to cause its silencing in prostate cancer cells [161]. Mitogen-activated protein kinase (MAPK) pathway activation is observed in nasopharyngeal and gastric cancer tissues [162,163]. Contrarily, Lf supplementation reduced cancer cell proliferation in vitro and impeded tumor growth in vivo in many murine models [164]. Moreover, higher levels of Lf are correlated to better prognosis in breast carcinoma patients [165]. Lf supplementation decreased colon cancer progression in humans [166] and significantly improved clinical prognosis in colorectal cancer patients receiving chemotherapy and in breast carcinoma patients [167]. Although it is unclear whether such tumor suppressive effects are mediated by its iron-binding role, Lf has emerged as a promising prognostic factor and adjuvant therapy.

**Figure 2:**
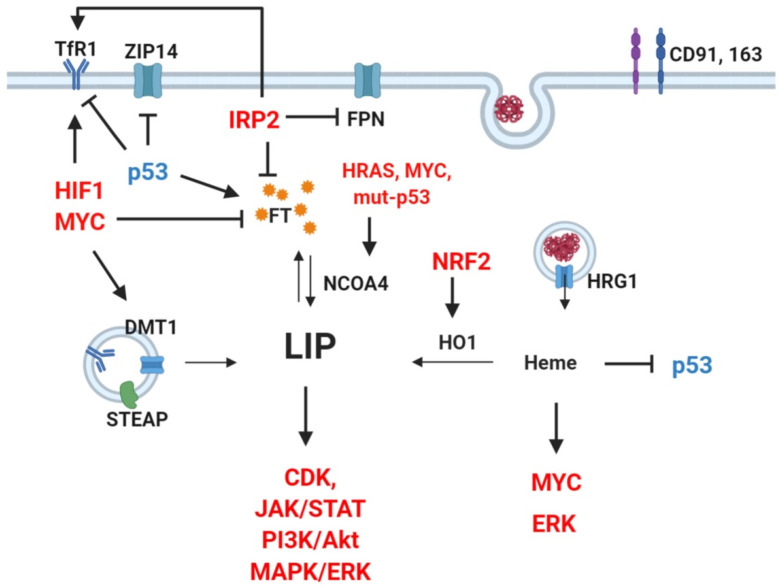
Crosstalk between oncogenes, signaling pathways, and iron regulators. Red: upregulation reported in cancer. Blue: downregulation reported in cancer. Several oncogenic transcription factors, signaling pathways, and proto-oncogenes directly regulate key players of iron metabolism. Notably, TfR1 upregulation can be caused by activation of HIF1 and MYC overexpression of IRP2, or loss of wild-type p53 [51,155,184,185]. Similarly, ferritin (FT) decrease can result from activation of MYC, IRP2, or p53 mutation [51,155,185]. Such a vicious cycle is sustained as increased LIP promotes CDK, JAK/STAT, PI3K, MAPK/ERK pathways [172], whereas heme can further enhance ERK and MYC and inhibits p53 [181,186,187].

TfR2 is commonly upregulated in human cancer cell lines as well [168,169], and was shown to act as a signaling protein activating the mitogen-activated protein kinase (MAPK) pathway in human leukemia cells [170]. Studies in glioblastoma and leukemia have established a correlation between TfR2 expression and tumor grade, but TfR2 overexpression also increased sensitivity to chemotherapy and, thus, is associated to favorable prognosis [169,171]. Other proteins involved in the uptake of iron that show abnormally high expression in cancers include DMT1 as well as heme importers HCP1, HRG1, CD91, and CD163, which are associated to dismal prognosis [42,172,173,174,175,176,177,178,179]. For instance, DMT1-imported iron was shown to activate cyclin-dependent kinase 1 (CDK1) and JAK1/STAT1, contributing to colorectal tumorigenesis, and DMT1 inhibitor impeded tumor growth in vivo [172]. CD91 was shown to promote migration and invasion through matrix metalloproteinase (MMP) induction in human glioblastoma cells and via extracellular signal-regulated kinase (ERK) pathway activation in human thyroid carcinoma cells [180,181]. Finally, a scarcely investigated yet noteworthy iron importer is ZIP14, whose main substrate is zinc but that can also mediate the uptake of NTBI and intracellular release of TBI from endosomes [182]. In liver cancer cells, knockdown of tumor suppressor p53 induced iron uptake through ZIP14, highlighting its potential implication in tumorigenesis [183] (Figure 2).

Lipocalin 2 (LCN2 or NGAL) is a soluble iron-binding glycoprotein expressed mainly by neutrophils [188]. Data about its role in cancer remain controversial and vary depending on iron saturation and on the cancer type. Interestingly, LCN2 can either import or export iron and, consequently, promotes proliferation or apoptosis [189,190,191]. A clear correlation between LCN2 levels and tumor grade was found in in breast and thyroid cancers, whereas the opposite was suggested in ovarian and pancreatic cancers [192]. Similarly, both LCN2 overexpression and knockout reduced orthotopic pancreatic cancer tumor growth in mouse models [193,194,195]. However, these results collectively indicate that LCN2 is also involved in angiogenesis and inflammation within the tumor.

Since iron uptake is strongly enhanced, it is not surprising that cancer cells present aberrant intracellular iron storage and trafficking in order to cope with increased risk of iron-related oxidative stress. Reduction of ferric to bioactive ferrous iron by STEAP reductases is required to release endosomal TBI to the cytosol. Particularly, STEAP 1 and 2 are overexpressed in various human cancers and were shown to drive cancer cell proliferation and resistance to apoptosis [196,197,198]. In addition to the abnormally high levels of circulating ferritin, some cancers exhibit alterations in both ferritin expression and intracellular localization. Variable clinical outcomes have been reported accordingly. For example, glioblastoma and breast cancer cells exhibited nuclear expression of FTL and FTH, respectively [199,200]. In the latter, nuclear expression of FTH was shown to prevent iron-derived oxidative DNA damage [200]. Although both FTH and FTL are upregulated in head and neck cancers, only FTH predicted negative prognosis [201,202], whereas the opposite was found in glioblastoma [199]. In melanoma cells, FTL is required to withstand oxidative stress and prevent apoptosis [203]. Coherently, knockdown of FTH increased sensitivity to apoptosis triggered by ROS in mesothelioma cells [204]. Of note, FTH overexpression has been linked to chemotherapy (doxorubicin and cisplatin)-resistance in breast and ovarian cancers [200,205]. Interestingly, p53 activation induced both FTH and FTL expression in human lung cancer cells [155]. NCOA4, which is responsible for ferritin degradation and cytosolic iron increase, was found overexpressed in transformed endometriotic cells and pancreatic cancer cell lines and correlated to prostate cancer risk [206,207,208]. Furthermore, inactivation of p53 and activation of transforming protein p21 (HRAS) and proto-oncogene MYC led to overexpressed NCOA4 [206]. Noteworthily, ferritin heavy chains can be imported by endocytosis after binding to TfR1 [209]. Scavenger receptor class A member 5 (SCARA5) was also shown to mediate ferritin uptake [210,211]. 

SCARA5 downregulation was found in human breast cancer tissues and cell lines and correlated with tumor size and metastatic potential. In the same in vitro models, overexpression of SCARA5 inhibited ERK, AKT, and Signal Transducer and Activator of Transcription 3(STAT3) pathways, impeded cell proliferation, migration, colony formation, and induced apoptosis [212]. Moreover, promoter hypermethylation was found to mediate the downregulation of SCARA5 in hepatocarcinoma cells and in human breast tumors [213]. Therefore, SCARA5 has been considered to play a role as a tumor suppressor, although its contribution to physiological iron metabolism is not fully clear. 

In leukemia and lung cancer cells, increased heme synthesis and availability also increased cell proliferation by enhancing mitochondrial oxidative metabolism [214,215,216]. Furthermore, recent studies have demonstrated that labile heme fosters cell proliferation by inhibiting p53 [187] and inducing MYC [186]. Interestingly, heme was shown to interfere directly with the p53 gene in vitro and in vivo while inducing proteosomal degradation of p53 protein [187]. Nevertheless, controversial studies exist when it comes to the effect of heme degradation by HO1 in cancer cells—some found that HO1 deficiency led to defective DNA, carcinogenesis, and resistance to ferroptosis (discussed hereafter); other data, however, indicated that HO1 induction promotes cancer cells antioxidant potential and cancer growth [217,218]. Notably, elevated HO1 activity induced matrix metalloproteinase 1 (MMP1) and stimulated migration and invasion in human breast cancer cells [219].

Despite sharing almost identical roles, IRPs show rather distinct patterns in tumors. Downregulation of IRP1 was found in hepatocellular carcinoma and predicted tumor stage and prognosis [172], and IRP1 overexpression in human non-small cell lung carcinoma cells suppressed tumor growth in mice [220]. In contrast, colorectal tumors showed IRP2 overexpression, which results from enhanced MAPK signaling and is associated with proto-oncogene B-Raf mutations [221]. In prostate cancer, IRP2 is predominantly upregulated, and decreased tumor growth due to apoptosis induction was observed after knockdown of IRP2, but not IRP1, in prostate cancer cell lines [222]. Similarly, although both IRPs show increased expression in breast cancer cells compared to non-malignant mammary epithelial cells, TfR1 and FT levels were altered only by IRP2 knockdown, which led to decreased tumor growth in vivo. Moreover, IRP2 expression correlated with histological grade and molecular subtype of human breast cancer. [223]. Thus, although both are potential prognostic factors, one IRP might prevail over the other without having the same expression trend, according to the cancer type. In cancer cells, other notable regulators of cellular iron metabolism include hypoxia-inducible factor 1α (HIF1α), the proto-oncogene MYC, and nuclear factor erythroid 2-related factor 2 (NRF2). In oxidative stress or iron overload conditions, NRF2 induces FTH, FTL, ferroportin, and heme oxygenase transcription to prevent excessive ROS formation promoted by iron availability [224]. Constitutive activation of NRF2 has been consistently found in various cancer types and linked to poor prognosis [225,226]. However, due to its pleiotropic effects, whether cancer progression promoted by NRF2 is iron-dependent or not has not been clear. In B cell lymphoma cells, MYC activates the transcription of TfR1 and DMT1 and represses that of FTH and FTL to increase the labile iron pool [185]. Similarly, hypoxia induces TfR1, DMT1, and hepcidin transcription through stabilization of HIF1α, leading to increased intracellular iron content [130,184,227].

In line with elevated hepcidin levels, decreased iron export is also a feature of several cancers [228,229,230,231]. In breast and pancreatic cancers, low FPN expression is associated with worse prognosis, while higher FPN expression in breast cancer patients corresponded to a cohort of patients presenting very high progression-free survival [231]. Coherently, overexpression of FPN in breast cancer cells showed decreased proliferation, colony formation, and tumor growth as well as liver metastases [230,232]. Likewise, FPN transcripts were downregulated in multiple myeloma cells isolated from patients compared to plasma cells from healthy donors and correlated with negative clinical outcomes. Furthermore, activation of STAT3 was found to mediate myeloma cell proliferation [233]. In several cell lines of prostate cancer, low FPN levels resulting from hepcidin upregulation were shown to promote proliferation, migration, and resistance to apoptosis [234], and FPN overexpression induced p53 and autophagy and reduced tumor growth in vivo [235]. Interestingly, human colorectal tumors were reported to upregulate FPN, but a histological analysis revealed aberrant cytoplasmic localization of the transporter—hence, non-functional FPN [236]. In summary, cancer cells commonly enhance iron import and suppress its export, while some uncertainty remains regarding how they cope with increased labile iron. 

## 4. Iron-Induced Oxidative Stress and Ferroptosis

Ferroptosis is an iron-dependent form of regulated cell death that occurs as a consequence of excessive lipid peroxidation [237]. The occurrence of ferroptosis essentially depends on the availability of redox active iron. Free iron participates directly in radical-generating reactions, notably Fenton’s reaction, in which ferrous iron reacts with hydrogen peroxide, yielding the hydroxyl radical (Fe^2+^ + H_2_O_2_ → Fe^3+^ + OH + OH^−^). ROS then trigger lipid peroxidation in the presence of unsaturated fatty acids, giving rise to a chain of oxidative reactions which propagate through the polyunsaturated fatty acids (PUFAs) of phospholipids constituting the cell membrane. The build-up of oxidized lipids ultimately leads to cell death [238]. Indeed, cells are equipped with anti-oxidant enzymes that neutralize lipid ROS to prevent further oxidative damage, notably glutathione peroxidase 4 (GPX4), which reduces oxidized lipids [239]. Beside iron chelators, lipophilic antioxidants, including vitamin E [240], ferrostatin-1 [241], and coenzyme Q10 (CoQ10, also known as ubiquinone) [242], have also been shown to prevent ferroptosis. Likewise, ferroptosis suppressor protein 1 (FSP1), previously known as AIFM2, was shown to protect cells from ferroptosis by catalyzing NAD(P)H-dependent regeneration of CoQ10, representing a parallel and synergistic pathway alongside GPX4-mediated lipid-ROS detoxification [242]. In contrast, ferroptosis is inducible in vitro by inhibiting GPX4 or by raising intracellular iron levels and ROS. Consequently, the expression levels of iron-regulating proteins, such as TfR1, FPN, or FT, determine the sensitivity of cancer cells to ferroptosis [243,244]. Similarly, increased ROS generation resulting from artesunate treatment caused ferroptotic death in PDAC cell lines expressing constitutively active KRAS [245]. Moreover, recent studies have found that cancer cells in a mesenchymal or drug tolerant persister state are more sensitive to GPX4 inhibitors and, hence, prone to ferroptosis [246,247]. An increasing number of studies have recently validated the efficacy of ferroptosis induction in reducing cell proliferation and tumor growth in vivo, either alone or in association with standard chemotherapy [248,249]. Although the role of ferroptosis in physiological conditions remains largely unknown, these studies underscore the promising clinical prospect of ferroptotic inducers as anticancer therapies.

## 5. Iron in the Tumor Environment 

A hallmark of malignant cells is the ability to reshape the environment where they reside, inducing angiogenesis, remodeling the extracellular matrix, and evading the host to sustain their growth [250]. Initially, immune cells recognize cancer cells as foreign and, therefore, they remodel their metabolism to compete for available iron with malignant cells [251]. Afterwards, chronic inflammation may result in evasion of immune surveillance by cancer cells, altering the immune cells toward an anti-inflammatory iron-releasing phenotype [252,253]. Polarization of tumor-associated macrophages (TAMs) determines their iron metabolism phenotype [253]: pro-inflammatory, M1-like TAMs exhibit upregulated FT levels coupled with decreased FPN, hence showing an iron-sequestering phenotype [254]. Interestingly, iron-loading in macrophages was associated with improved survival in lung cancer patients [255]. In contrast, anti-inflammatory, M2-like TAMs instead release iron and support tumor growth [256]. In particular, FPN is not the only route of iron secretion by M2-like TAMs, as its knockdown did not alter iron levels in breast cancer cells, but also, LCN2 expression mediates iron secretion by macrophages in the tumor microenvironment [257]. Iron-loading in TAMs can directly contribute to tumor growth, by feeding iron to cancer cells, or indirectly, by fostering angiogenesis. For instance, LCN2 expression by TAMs promotes lymphangiogenesis and metastasis progression in a murine breast cancer models [258]. Concerning angiogenesis, the effect of iron seems different according to the dose. While iron supplementation inhibited vascular endothelial growth factor (VEGF) signaling in endothelial cells in vitro and reduced lung carcinoma vascularization in vivo [259], LCN2 supplementation resulted in ROS accumulation and increased brain endothelial cell migration which was reversible upon iron chelation [260]. Hence, low dose of iron can inhibit angiogenesis, whereas high dose promotes oxidative stress that is widely recognized to promote angiogenesis [261].

Finally, cancer-associated fibroblasts (CAF) can also alter tumor iron metabolism, as fibroblast IL-6 secretion was shown to foster cancer cell hepcidin expression which supported iron retention in cancer cells in breast cancer spheroids [262].

Iron metabolism in the tumor microenvironment is an emerging field of research, and beside the complexity of its role in all the different cell populations involved, an in-depth understanding of how iron is handled in its niche will potentially provide novel anticancer strategies to interfere with iron metabolism at microenvironment level.

## 6. Effects of Iron Supplementation or Chelation on Tumorigenesis

Accumulating studies have identified iron as a relevant promoter of tumorigenesis in different aspects, including genetic and epigenetic alterations, tumor initiation, cell motility, and invasiveness; hence, a large pool of data supports iron deprivation as a therapeutic strategy in cancers.

Repeated administration of iron led to high incidence of renal cell carcinoma in rats, which was associated with large-scale genomic modifications, presumably due to increased oxidative stress [263]. Similarly, iron supplementation induced hypomethylation of oncogenes involved in PI3K/AKT, MAPK/ERK, and RAP1/RAS pathways in colonocytes [264]. In vitro, treatment with iron triggered increased migration and invasion via ROS production in human lung carcinoma and melanoma cells [265]. Treatment of colon cancer cells with ferric chloride induced an aggressive, mesenchymal phenotype with loss of intercellular adhesion by E-cadherin, which could be rescued by iron chelation with deferoxamine (DFO) [266]. Moreover, DFO reduced histone demethylases in human breast cancer cells and raised their sensitivity to chemotherapies in vitro [267]. In line with these findings, iron deprivation in vitro by iron chelators led to cell cycle inhibition and apoptosis of human colon, liver, and breast cancers and neuroblastoma, notably through activation of p53 and cyclin-dependent kinases [187,235,268]. High-throughput screening further identified iron chelators DFO, deferasirox, and ciclopirox as inhibitors of Wnt/β-catenin signaling, which is pivotal to cancer initiation and maintenance [269]. DFO was also shown to repress stem-like properties, including markers of stem-like cells and sphere-forming ability, in cholangiocarcinoma cells [270]. However, DFO-induced iron deficiency also led to normoxic stabilization of hypoxia-inducible factor-1α as well as epithelial-to-mesenchymal transition (EMT) in colorectal cancer cells, both of which have been linked to increased tumor growth [271]. In conclusion, in addition to the evident roles of iron in essential processes, such as cell respiration and genome replication, these findings highlight the multiple facets of iron as a peculiar metabolic cofactor and signaling element as well as initiator of oxidative stress and indicate, altogether, that modulating iron levels can impact different steps of tumorigenesis. 

## 7. Clinical Application of Iron Chelation/Normalization Therapies

The phenotype of iron addiction in cancer cells represents, indeed, a therapeutic target of particular interest. Targeted therapies currently under commercial development are mainly aiming at TfR1 normalization or antagonism. Strategies including monoclonal antibodies, targeting peptides, and liposomal plasmid-based gene therapy have been proposed as treatments for various solid tumors [48,272]. Many have entered clinical trials, but limited data are available up to date. On the other hand, iron chelators, such as deferoxamine (DFO), deferasirox (DFX), and deferiprone (DFP), have been successively introduced to the market over recent decades, initially as treatments for iron overload conditions [48]. In recent years, their well characterized pharmacokinetic properties and promising in vitro anti-cancer effects have prompted the initiation of multiple preclinical studies and, eventually, clinical studies. In particular, DFO and DFX have shown efficacy alone or as adjuvants to chemotherapy in human xenografts of gastric, esophageal, pancreatic, liver, and breast cancers in mice [273,274,275,276,277]. However, in patients presenting advanced stages of hepatocarcinoma, DFX did not impact tumor progression and increased anorexia and serum creatinine [276]. Interestingly, DFP not only chelates LIP but also promotes intracellular ROS production and single-strand DNA breaks at lower doses in human hepatocarcinoma cells in vitro [278] and was recently found to reduce orthotopic prostate tumor growth in mice [279]. Other chelators that are less selective and specific are ciclopirox olamine, tachpyridine, and thiosemicarbazones, as well as epigallocatechin, curcumin, and silybin—three natural compounds that have remarkably broad activities. Ciclopirox olamine is a fungicide that initially showed promising effects in different mouse models [280,281,282,283] and in human hematological malignancies but showed a very poor pharmacokinetic profile (low solubility and rapid metabolism and clearance) [284]. A prodrug was consequently developed and was shown to be effective in urothelial cancers in rats and dog and is now under a phase I clinical trial for advanced solid tumors [285] (NCT03348514). Similar to DFP, tachpyridine also depletes intracellular iron while inducing oxidative stress [286]. Beside iron, it also binds zinc and copper; nevertheless, tachpyridine owes its cytotoxicity predominantly to its iron-chelating ability [287]. Thiosemicarbazones constitute another class of iron chelator that present ideal pharmacokinetic features, with the most prominent one being triapine. The latter showed efficacy in leukemia and ovarian and lung cancers and could even cross the blood–brain barrier; hence, it would be a potential candidate for the treatment of brain metastases [109]. Despite the observation of increased methemoglobin levels with high doses of triapine, results from a phase II trial proved its efficacy as an adjuvant therapy (with cisplatin) in advanced uterine cervix or vaginal cancers without significant toxicity and led to a phase III trial [288] (NCT02466971). Collectively, adjuvant therapy targeting iron metabolism will likely be available as an anticancer therapeutic option in the near future. 

## 8. Concluding Remarks

Although iron is one of the most abundant elements on Earth, it constitutes a rate-limiting factor for physiological metabolism to sustain life. In particular, cancer cells rely heavily on the availability of iron and commonly exhibit striking alterations of iron metabolism, which have been shown to directly contribute to disease progression. Indeed, iron as a cofactor enables cell respiration, protein translation and even DNA replication and repair—required to sustain tumor growth. Furthermore, the tumor microenvironment can also reprogram iron metabolism and, accordingly, either cope with or fight against malignant cells. Accumulating clinical data indicate that such alterations in iron metabolism can serve as diagnostic tool and even determine prognosis. Importantly, the increased avidity of cancer cells for iron is a potential target for therapy, as it might be exploited as a trojan horse to burst oxidative stress and induce ferroptosis. Modulating iron levels or its metabolism has shown promising outcomes in vitro, and some strategies have even reached success in early phases of clinical trials as adjuvant in anticancer therapies. However, as iron is essential to all types of cells, targeting malignant cells with selectivity remains tricky, and chronic administration of iron chelators might induce systemic iron deficiency, which is also deleterious. Understanding the mechanisms to sensitize cancer cells to iron chelation or overload and targeting the tumor microenvironment will provide further progress to the development of iron-targeted anticancer strategies.

## Abbreviations

ABCB7	ATP-Binding Cassette sub-family B member 7
ACO	Aconitase
BMP	Bone-Morphogenetic Protein
CAF	Cancer-Associated Fibroblast
CAT	Catalase
CD	Cluster of Differentiation
CDK1	Cyclin-Dependent Kinase 1
CoQ10	Coenzyme Q10
COX	Cyclooxygenase
CP	Ceruloplasmin
DFO	Desferoxamine
DMT1	Divalent Metal Transporter 1
DOHH	Deoxyhypusine Hydroxylase
Dycb	Duodenal cytochrome b
EMT	Epithelial to Mesenchymal Transition
ETC	Electron Transport Chain
ERFE	Erythroferrone
ERK	Extracellular signal Regulated Kinase
FBXL15	F-Box and Leucine Rich Repeat Protein 15
FSP1	Ferroptosis Suppressor Protein 1
FTX	Frataxin
FT	Ferritin
FTH	Ferritin Heavy Chain
FTL	Ferritin Light Chain
FPN	Ferroportin
GPX4	Glutathione Peroxidase 4
HCP1	Heme Carrier Protein 1
HRG1	Heme-Responsive Gene 1
HEPC	Hepcidin
HEPH	Hephestin
HIF1	hypoxia-inducible factor 1
HO1	Heme Oxygenase 1
IL	Interleukin
IRE	Iron Responsive Element
IRP1/2	Iron Regulatory Protein 1/2
ISC	Iron-Sulfur Cluster
ISCU	ISC Assembly Enzyme
JAK	Janus Kinase
KRAS	Ki-ras2 Kirsten rat sarcoma viral oncogene homologue
LCN2	Lipocalin2
Lf	Lactoferrin
LIP	Labile Iron Pool
MAPK	Mitogen Activated Protein Kinase
MFRN	Mitoferrin
MMP	Matrix Metallo-Proteinase
NAF1	Nutrient-deprivation Autophagy Factor-1
NCOA4	Nuclear Receptor Coactivator 4
NEET	CDGSH iron-sulfur domain-containing protein
NFKB	Nuclear Factor Kappa-light-chain-enhancer of activated B cells
NFS1	Cysteine Disulfurase
NO	Nitric Oxide
NOS	NO Synthase
NRF2	Nuclear Factor Erythroid 2-Related Factor 2
NTBI	Non-Transferrin Bound Iron
PI3K/Akt	Phosphatidylinositol 3-Kinase/protein kinase B
PHD	Prolyl Hydroxylase
PDAC	Pancreatic Ductal Adenocarcinoma
RBC	Red Blood Cells
ROS	Reactive Oxygen Species
SCARA5	Scavenger receptor class A member 5
SDH	Succinate Dehydrogenase
STAT	Signal Transducer and Activator of Transcription
STEAP	Six-Transmembrane Epithelial Antigen of Prostate
TAM	Tumor-Associated Macrophages
Tf	Transferrin
TfR	Transferrin receptor
TBI	Transferrin-Bound Iron
TCA cycle	Tricarboxylic Acid cycle/ Krebs cycle
TNF-α	Tumor Necrosis Factor-α
VEGF	Vascular Endothelial Growth Factor
XOR	Xanthine Oxido-Reductase
